# A 14-year-old boy with multiple trauma and bilateral basal ganglia hemorrhage due to coronavirus disease 2019: a case report

**DOI:** 10.1186/s13256-023-03824-1

**Published:** 2023-03-10

**Authors:** Payman Asadi, Saba Maleki, Seyyed Mahdi Zia Ziabari, Nazanin Noori Roodsari

**Affiliations:** 1 School of Medicine, Guilan University of Medical Sciences (GUMS), Rasht, Guilan Province Iran; 2 Roud Trauma Research Center, Guilan University of Medical Sciences, Rasht, Iran; 3 Clinical Research Development Unit of Poursina Hospital, Department of Emergency Medicine, School of Medicine, Guilan University of Medical Sciences, Rasht, Iran; 4 Department of Emergency Medicine, School of Medicine, Guilan University of Medical Sciences, Rasht, Iran

**Keywords:** COVID-19, Basal ganglia hemorrhage, Intracranial hemorrhage, Neurological manifestations, Neuroimaging

## Abstract

**Background:**

In December 2019, coronavirus disease 2019 spread worldwide, causing acute respiratory distress syndrome. Coronavirus disease 2019 presents from an asymptomatic infection to severe disease causing multiorgan failure. Neurological manifestations were observed in some patients, including intracerebral hemorrhage. Bilateral basal ganglia hemorrhage is rare due to trauma.

**Case presentation:**

Our patient was a 14-year-old Iranian boy with multiple trauma and loss of consciousness who tested positive for coronavirus disease 2019. The brain computed tomography scan reported bilateral basal ganglia hemorrhage. Bilateral ground glass opacity was reported through a chest computed tomography scan.

**Discussion and conclusions:**

In this study, we reported a 14-year-old boy referred to the emergency room due to multiple trauma. Through the medical interventions, bilateral basal ganglia hemorrhage was discovered incidentally. Coronavirus disease 2019 was detected in this patient on the basis of findings in chest computed tomography scan and positive real reverse transcription polymerase chain reaction test. Several clinical reports and series exploring the relationship between coronavirus disease 2019 and ischemic strokes have been published. Coronavirus disease 2019, like other acute respiratory syndromes, can invade the central nervous system through hematogenous and neuronal dissemination or it can be an immune response to the cytokine storm. In conclusion, it is vital to know the pathophysiology of the neurological manifestations of coronavirus disease 2019 and prevent the mild neurological manifestations leading to severe conditions.

## Introduction

Severe acute respiratory syndrome–coronavirus-2 (SARS–CoV-2) caused coronavirus disease 2019 (COVID-19) in Wuhan, China in December 2019. COVID-19 is a contagious respiratory disease spreading worldwide [1]. COVID-19 can appear from an asymptomatic situation to acute respiratory distress syndrome (ARDS) and eventually multiorgan damage [2]. The central nervous system (CNS) can be involved in COVID-19. Some neurological manifestations of COVID-19 include dizziness, headache, hypogeusia, hyposmia, ataxia, seizure, ischemic stroke, cerebral hemorrhage, encephalopathy, encephalitis, meningitis, seizure, cerebral vein thrombosis, and Guillain–Barre syndrome [3]. Intracranial hemorrhage (ICH) is an uncommon situation due to COVID-19. This may happen because of some risk factors such as arterial hypertension or anticoagulant therapy [2]. Expression of angiotensin-converting enzyme 2 (ACE2) in glial cells and neurons may be a possible etiology for the neurological manifestation of COVID-19 [4]. In this case study, we will report bilateral basal ganglia hemorrhage in a 14-year-old boy who tested positive for COVID-19 and denied any past medical history or anticoagulant consumption.

## Case presentation

The case is a 14-year-old Iranian boy with no past medical history presenting with multiple trauma from a motorcycle accident who was transferred to the Poursina hospital emergency room (ER) in Rasht on 9 June 2020. In addition, he had an extensive laceration on his right knee and a star-shaped laceration on his parietal region. O_2_ saturation was 93%, temperature was 38.5 °C, and the other vital signs were stable. In the ER, his Glasgow Coma Scale (GCS) score was 3/15 (E1V1M1). No free fluid in the abdomen and pelvic cavity in focused assessment with sonography for trauma was reported. A right bundle branch block was observed in his electrocardiogram (ECG). Echocardiography was performed and reported as normal. He was then referred to the neurosurgery ward and was hospitalized for almost 40 days.

**Fig. 1:**
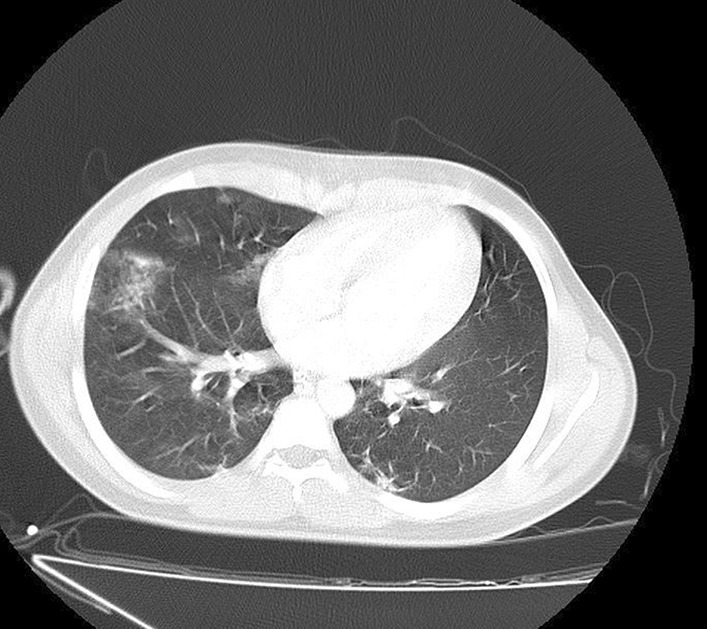
Patchy ground-glass opacities in both lungs, especially in the right lung, propounded by COVID-19 infection. Heart size is normal. Pleural effusion is not seen. Chest wall is normal

He had no signs and symptoms related to COVID-19. However, during hospitalization, spiral high-resolution computed tomography (HRCT) scan of his lung without contrast revealed patchy ground-glass opacity in both lungs (mostly in the right lung) (Fig. 1). These findings suggested that the patient was infected by COVID-19. Finally, the COVID-19 infection was confirmed by consecutive positive reverse transcription polymerase chain reaction (RT-PCR) test.

**Fig. 2:**
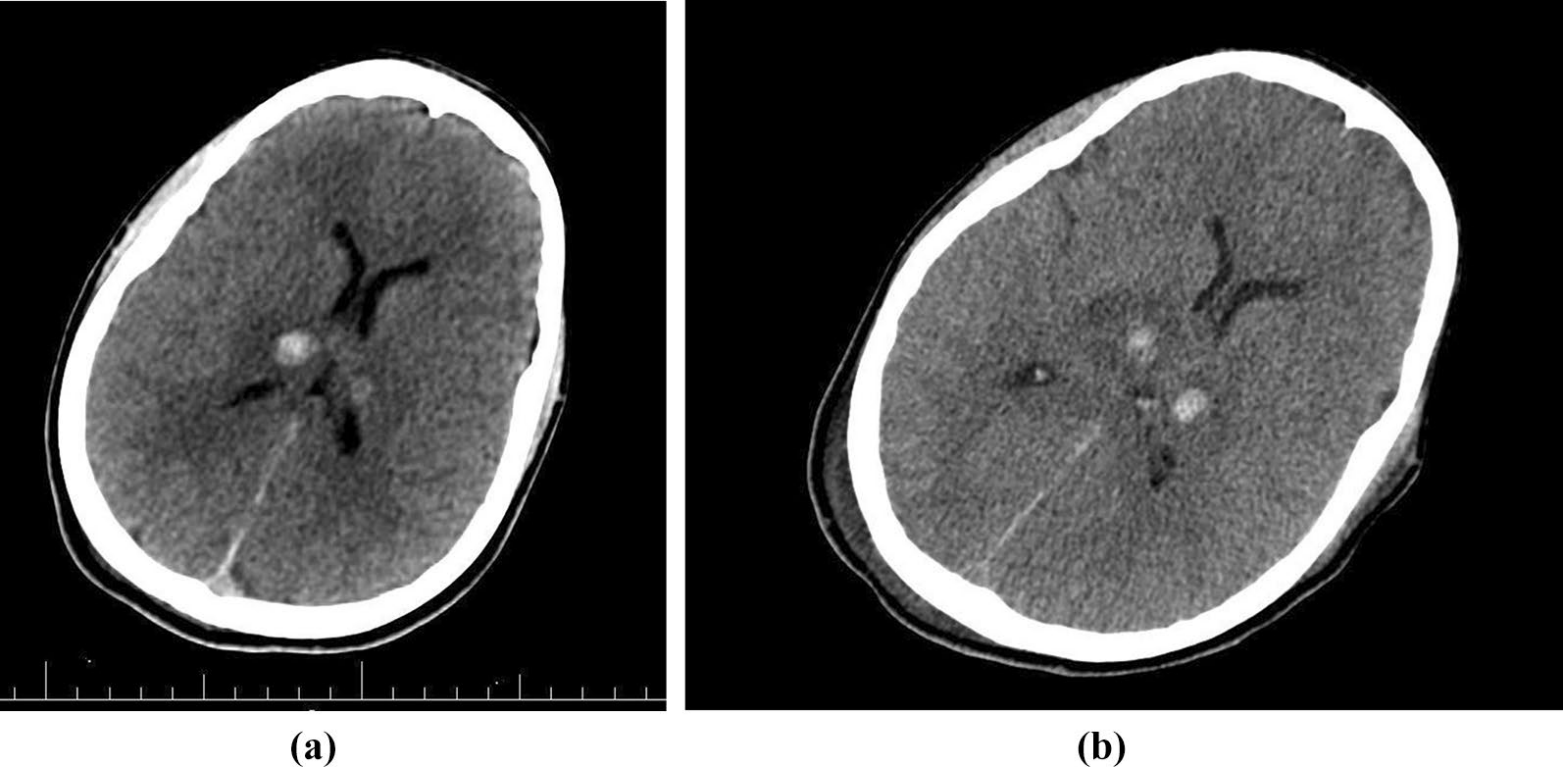
**A** CT scan of the brain at the ER showing two hyperdense lesions in the thalamus and parenchymal edema. **B** Brain CT scan taken after a few days, showing no midline shift, and ventricular edema less than previous CT scan

Following a brain CT scan, a deep intracranial hemorrhage in the thalamus was found (Fig. 2A). The contusion of the right parietal lobe without hydrocephalus and midline shifts were reported (Fig. 2B). Furthermore, no spinal fractures and displacements were reported in his cervical spine CT scan. Abdominal and pelvic CT scan were also normal.

His laceration of the parietal region was repaired by surgery. In addition, the repair and debridement of the right knee laceration were carried out. Afterward, he was intubated and a collar fixed his cervical spine. He was treated with lasix and mannitol because of raised intracranial pressure. After supportive therapy for almost 40 days, he was discharged after educating his parents about his care at home, while his GCS was 6/15 (E2V1M3).

## Discussion

In this study, we reported a 14-year-old boy referred to the ER due to multiple trauma. He had no history of coagulopathy or anticoagulant consumption. In this case, trauma precipitated parietal contusion. Through medical interventions, bilateral basal ganglia hemorrhage was discovered incidentally.

Several clinical reports and series exploring the relationship between COVID-19 and ischemic strokes have been published [5].

Neurologic symptoms in COVID-19 can be divided into several categories, ranging from nonspecific symptoms such as a headache to severe forms such as cerebrovascular disease [6].

Basal ganglia hemorrhage due to traumatic events is rare [7].

Systemic and metabolic disease, neurodegenerative disease, and vascular disease can involve basal ganglia and thalamus [8, 9].

COVID-19, like other acute respiratory syndromes, can invade the central nervous system (CNS) through hematogenous and neuronal dissemination, or it can be an immune response to the cytokine storm [10, 11].

**Fig. 3:**
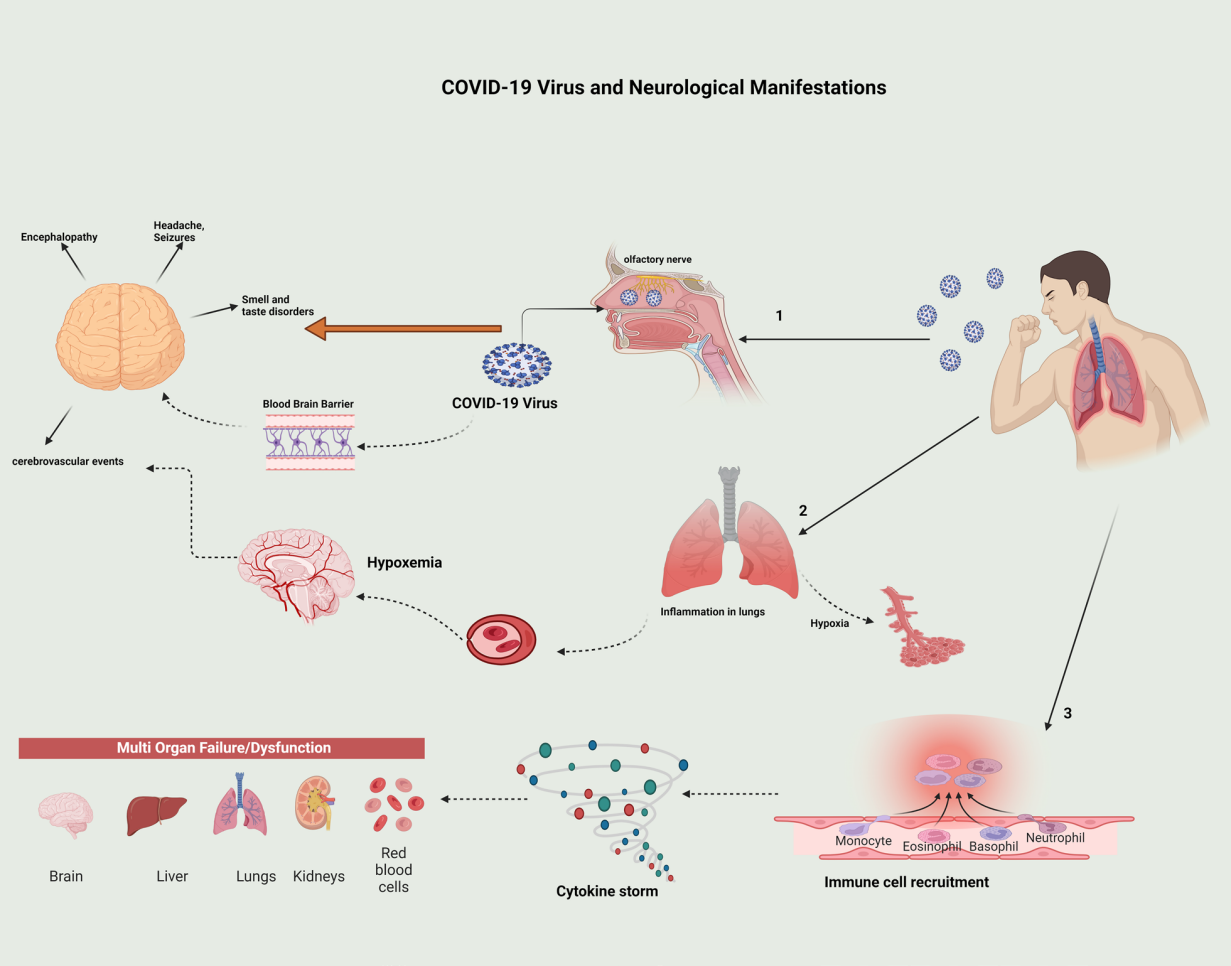
Representation of how COVID-19 can cause neurological manifestations such as headache, seizure, smell and taste disorders, encephalopathies, and cerebrovascular events

There is not enough data about the underlying mechanisms of neurological disorders caused by COVID-19. A total of 2% of patients have neurological symptoms. One of the ways in which COVID-19 can invade the neurological system is through the mucous layer of the nose that contains the olfactory receptor cells. Olfactory nerves might deliver viruses and infections to the brain. On the other hand, COVID-19 causes respiratory infection and the level of oxygen in blood may decrease. Brain function can be impaired through hypoxemia (Fig. 3) [12].

Some other pathophysiology of ischemic stroke in COVID-19 are hypoperfusion of the brain, hypertension leading to posterior reversible encephalopathy syndrome, and septic embolization in patients with bacterial superinfection (Fig. 3) [13].

ACE2 serves important roles in various parts of the body, such as the lung, kidney, brain, liver, and endothelial cells. COVID-19 decreases the expression of ACE2. Due to reduced ACE2 levels, the renin–angiotensin–aldosterone system (RAAS) will deteriorate and cause multiorgan damage. Diminished ACE2 can cause intracerebral hemorrhage through several mechanisms. For instance, increasing the blood pressure, endothelial dysfunction, damage to the brain vessels, and finally depression of angiotensin-(1-7) and Mas receptor [Ang (1-7)/MasR] signaling, which has a neuroprotective, antifibrotic, and vasodilatory effect [14, 15].

Immune response to the cytokine storm and chemokines can disrupt the blood–brain barrier and neuroinflammation. The breakdown of the blood–brain barrier and inflammation both have a main role in ischemic stroke [16, 17].

Intracranial hemorrhage was documented in some cases as a complication of COVID-19. Some risk factors are arterial hypertension and anticoagulation therapy (pretreatment of unrelated disease to COVID-19 thromboprophylaxis) [2].

Hyperinflammatory state may be the main cause of complication in patients with diabetes mellitus (DM) as well as COVID-19. A case series was reported in October 2020 that included three patients with COVID-19 and ICH. All three patients had risk factors for ICH such as hypertension and DM. DM and ICH are associated through some mechanisms. High blood glucose can cause endothelial dysfunction. Dysfunction of the brain’s small vessel endothelial can lead to ICH [14].

There is a hypothesis that the state of hyperinflammation in diabetic patients is related to higher mortality in these patients [18]. High C-reactive protein (CRP) levels are a risk factor for stroke. High CRP levels may lead to ICH in patients with COVID-19 [19].

We reported a 14-year-old boy with bilateral basal ganglia hemorrhage and documented COVID-19. Our patient had no special past medical history of hypertension, DM, and other diseases. There is no evidence that he consumed anticoagulant agents before. There is some research that ICH is propounded as a complication of COVID-19. Basal ganglia hemorrhage related to trauma is rare [7].

The underlying pathophysiology for COVID-19 related ICH can involve hyperinflammatory state due to increased cytokine production. Although a few case series and case reports were published on some patients COVID-19 as well as ICH, more investigation and research regarding the association between COVID-19 and ICH are needed.

## Conclusions

It is essential to discover the pathophysiology of neurological manifestations of COVID-19. Neurological findings of COVID-19 range from mild conditions such as headache to severe conditions such as stroke. Therefore, acknowledgement as to the causes and prevention of the situations leading to critical events should be considered in treatments of COVID-19.

## Data Availability

The datasets obtained and analyzed in the current study are available from the corresponding author upon reasonable request.

## Abbreviations

COVID-19: Coronavirus disease 2019
CT scan: Computed tomography scan
ICH: Intracranial hemorrhage
GCS: Glasgow Coma Scale
ECG: Electrocardiogram
RT-PCR: Reverse transcription polymerase chain reaction
CNS: Central nervous system
DM: Diabetes mellitus
CRP: C-reactive protein
ER: Emergency room
ARDS: Acute respiratory distress syndrome
ACE2: Angiotensin-converting enzyme 2
HRCT: High-resolution CT scan
RAAS: Renin–angiotensin–aldosterone system
Ang(1-7)/MasR: Angiotensin-(1-7) and Mas receptor
